# [Retracted] Remodeling of energy metabolism by a ketone body and medium-chain fatty acid suppressed the proliferation of CT26 mouse colon cancer cells

**DOI:** 10.3892/ol.2026.15860

**Published:** 2026-09-14

**Authors:** Yui Kadochi, Shiori Mori, Rina Fujiwara-Tani, Yi Luo, Yukiko Nishiguchi, Shingo Kishi, Kiyomu Fujii, Hitoshi Ohmori, Hiroki Kuniyasu

Oncol Lett 14: 673–680, 2017; DOI: 10.3892/ol.2017.6195

Following the publication of this paper, it was drawn to the Editor's attention by a concerned reader that, regarding the mRNA expression data shown in Fig. 1 on p. 674, the Mct1 and Scot blots bore a striking similarity to each other, albeit with vertical and horizontal resizing; furthermore, data in this figure appeared to be strikingly similar to blots which were subsequently published in a pair of articles written by members of the same research group in the journals *Oncotarget* and *Cancer Science*, albeit where the experimental conditions were reported to be different. In addition, the microscopic data featured in Fig. 4E on p. 678 were remarkably similar to data that were included in a paper featuring many of the same authors that was published during the same year in the journal *World Journal of Gastroenterology*, where the experimental conditions were likewise reported to be different. A subsequent investigation of the data in this paper revealed that certain of the blots may also have been internally duplicated within Fig. 5A and C.

Given the identification of the abovementioned cases of data duplication and re-use across a range of publications, the Editor of *Oncology Letters* has decided that this paper should be retracted from the Journal on account of a lack of confidence in the presented data. The authors were asked for an explanation to account for these concerns, but the Editorial Office did not receive a reply. The Editor apologizes to the readership for any inconvenience caused.

